# Competencies in head and neck surgery teaching for specialty residents. Position statement of Brazilian Head and Neck Surgery Society

**DOI:** 10.1016/j.bjorl.2025.101710

**Published:** 2025-09-13

**Authors:** Rogério Aparecido Dedivitis, Mario Augusto Ferrari de Castro, Fátima Cristina Mendes de Matos, Marianne Yumi Nakai, Leandro Luongo de Matos, Marco Aurélio Vamondes Kulcsar, Carlos Takahiro Chone, Luiz Paulo Kowalski, Fernando Luiz Dias, André Cruz Martins, Bruna Carteiro Silva

**Affiliations:** a Faculdade de Medicina da Universidade de São Paulo (FMUSP), Departamento de Cirurgia de Cabeça e Pescoço, São Paulo, SP, Brazil; b Universidade Metropolitana de Santos, Faculdade Medicina, Departamento de Otorrinolaringologia e Cirurgia de Cabeça e Pescoço, Santos, SP, Brazil; c Universidade Estadual de Pernambuco, Departamento de Cirurgia de Cabeça e Pescoço, Recife, PE, Brazil; d Faculdade de Ciências Médicas da Santa Casa de São Paulo, Departamento de Cirurgia de Cabeça e Pescoço, São Paulo, SP, Brazil; e Universidade de Campinas (UNICAMP), Departamento de Otorrinolaringologia e Cirurgia de Cabeça e Pescoço, Campinas, SP, Brazil; f Instituto National do Cancer, Departamento de Cirurgia de Cabeça e Pescoço, Rio de Janeiro, RJ, Brazil; g Universidade Metropolitana de Santos, Faculdade Medicina, Santos, SP, Brazil

**Keywords:** Education, Medical, Competencies, Clinical, Residency and internship, Delphi method

## Abstract

**Objectives:**

To standardize the necessary competencies for Head and Neck Surgery residents related to the specialty.

**Methods:**

The Delphi process was employed in its sequential phases: the selection of an expert panel; a structured questionnaire containing a preliminary list of potential competencies; the electronic distribution of the questionnaire to the experts, with options to “maintain”, “remove”, or “modify” the competencies and to suggest the inclusion of new competencies; a second round indicating “agree” or “disagree” for each reformulated or new competency; and the final consensus.

**Results:**

Forty-six competencies were established for Head and Neck Surgery residents in the specialty.

**Conclusion:**

Head and Neck Surgery residents and fellows should be trained in depth for the competency framework encompassing both routine and complex surgical interventions.

**Level of evidence:**

5.

## Highlights

• A modified Delphi method with 25 experts was used to reach consensus.
• 46 competencies were approved with at least 90% agreement.
• Competency-based training is essential for preparing surgeons.

## Introduction

Contemporary medical education faces significant challenges, primarily related to the effective integration of theoretical and practical clinical competencies that respond to the dynamic demands of the medical profession. Within this context, the establishment of minimum competencies emerges as a guiding framework in Medical Education, aligning teaching with the actual requirements of clinical practice. Competencies represent specific tasks or responsibilities that learners must demonstrate the ability to perform independently upon reaching designated proficiency levels.^1^^,^^2^

In the specific domain of Head and Neck Surgery, given the high prevalence of diverse benign and malignant conditions affecting this anatomical region. Head and Neck Surgery residents must master a comprehensive array of competencies extending beyond theoretical medical knowledge. Consequently, specialized knowledge and technical skills in head and neck surgery are indispensable for future professional practice.^3^

In Head and Neck Surgery residency programs, competencies are essential for developing the technical proficiency and clinical judgment required to perform complex surgeries and high-precision procedures. Competence in tasks such as removing tumors from anatomically challenging locations, tissue reconstruction, and managing postoperative complications is critical, and repeated practice of these trusted activities is fundamental to forming a competent and autonomous surgeon.^4^

The teaching of Head and Neck Surgery in specialty residency/fellowship must provide adequate training for the new specialist. Therefore, the objective of this study is to formulate standardized competencies necessary for Head and Neck Surgery residents relating to their specialty training.

## Methods

This is a qualitative cross-sectional study to determine competencies related to Head and Neck Surgery for specialty residents at the completion of their training period. Data were collected through electronic questionnaires. The questionnaires were distributed online to professors and heads of Head and Neck Surgery residency training programs accredited by the Brazilian Society of Head and Neck Surgery. The responses received from the questionnaires were organized and evaluated using the Delphi methodology, seeking to reach consensus.

We employed a modified Delphi consensus approach to define the core competencies required for Head and Neck Surgery specialty residents.^5^ This systematic method has demonstrated robust reliability in establishing educational standards across surgical specialties through structured expert consultation.

The development of a comprehensive competency framework for head and neck surgery residency training necessitated a structured consensus process capable of synthesizing expert opinion across the field. We implemented a modified Delphi technique ‒ a methodological approach particularly suited for establishing educational standards in specialized surgical disciplines where objective evidence alone may be insufficient.

Our consensus panel comprised program directors and educational leaders from head and neck surgery departments affiliated with the Brazilian Society of Head and Neck Surgery. Panel members were rigorously selected based on predefined criteria emphasizing longitudinal experience in resident education, surgical expertise, and leadership within the specialty, ensuring balanced representation across geographic regions and institutional settings.

The consensus process was executed in a systematic, iterative manner. Initially, we extensively analyzed international training guidelines and contemporary literature on surgical competency development to construct a preliminary competency framework specific to head and neck surgery residency. This draft framework was subsequently distributed to panel members via a secure electronic platform.

In the first consensus round, experts evaluated each competency statement using a tripartite categorical system (maintain/remove/modify). When selecting “modify”, participants provided explicit recommendations for refinement. Additionally, the survey instrument incorporated dedicated sections for experts to propose novel competencies not represented in the initial framework but deemed essential for subspecialty practice.

Following the first round, we comprehensively tabulated expert responses, tabulated agreement percentages for each competency, and performed thematic analysis of qualitative feedback. This analytical process informed the construction of a reconfigured competency framework incorporating panel-suggested modifications and additions.

The second consensus round presented the revised framework to the expert panel using a binary response format (agree/disagree) supplemented by an optional commentary field for each competency. This methodological approach aligns with established best practices for terminal consensus determination in Delphi studies following exploratory initial rounds.

After the second round, a 90% agreement threshold was established for definitive consensus ‒ an intentionally rigorous criterion designed to ensure that only unambiguously essential competencies were incorporated into the final educational framework.

Throughout both rounds, strict anonymity of individual responses was maintained, eliminating potential influence from opinion dominance or hierarchical considerations ‒ a critical methodological feature of Delphi studies that enhances the validity of consensus outcomes. The entire research protocol was approved by the institutional ethics committee before implementation.

The entire process was conducted with anonymity among participants, a fundamental characteristic of the Delphi methodology that minimizes the influence of hierarchical or political factors in decisions.^6^

## Results

The expert consultation process involved 25 specialists who participated throughout all phases of the Delphi methodology. During the initial evaluation round, 44 potential competencies were presented for assessment. Of these, 30 received unanimous endorsement, while 14 required modifications based on expert feedback. Notably, no competencies from the initial proposal were recommended for removal. The panel additionally proposed two new competencies for consideration. Following comprehensive revision, the second-round evaluation demonstrated complete consensus (100% agreement) for 41 competencies, strong support (95% agreement) for four competencies, and substantial agreement (90%) for one competency, culminating in a final framework of 46 essential competencies for Head and Neck Surgery specialists, as detailed in Table 1.

**Table 1:** Head and neck surgery residency competencies.

#	Competency
1	Act with ethics and professionalism
2	Communicate gently, effectively, and empathetically with patients, family members, and other professionals
3	Work appropriately in a team
4	Conduct thorough medical history for patients with head and neck conditions
5	Perform physical examination of patients
6	Identify, evaluate, and investigate patients with head and neck conditions
7	Identify conditions requiring biopsy
8	Develop a care plan for patients with head and neck conditions
9	Perform postoperative dressing changes
10	Remove surgical drains
11	Change tracheostomy tubes
12	Perform closure of surgical incisions
13	Evaluate, investigate, and develop an action plan for patients with acute airway obstruction
14	Evaluate, investigate, and develop an action plan for patients with dysphagia or swallowing disorders in collaboration with a multidisciplinary team
15	Manage hospitalized patients in the service
16	Manage postoperative complications or adverse events
17	Evaluate, investigate, and develop an action plan for patients with dysphonia in collaboration with a multidisciplinary team
18	Evaluate, investigate, and develop an action plan for patients with head and neck mucosal cancer
19	Evaluate, investigate, and perform deep cervical drainage in patients with cervical abscess
20	Evaluate, investigate, and biopsy patients with suspicious cervical lymphadenopathy
21	Perform tracheostomy in intubated patients
22	Perform tracheostomy and/or cricothyroidotomy in patients with acute airway obstruction
23	Evaluate, investigate, and operate on patients with thyroid gland disorders
24	Evaluate, investigate, and operate on patients with parathyroid gland disorders
25	Evaluate, investigate, and operate on patients with salivary gland disorders
26	Evaluate, investigate, and operate on patients with benign or malignant skin lesions of the head and neck
27	Evaluate, investigate, and operate on patients with benign or malignant lip lesions
28	Evaluate, investigate, and operate on patients with congenital head and neck conditions
29	Perform reconstruction with local and regional flaps
30	Assist in reconstruction with microsurgical flaps
31	Evaluate, investigate, and operate on patients with benign or malignant oral cavity lesions
32	Evaluate, investigate, and operate on patients with benign or malignant pharyngeal lesions
33	Evaluate, investigate, and operate on patients with benign or malignant laryngeal lesions using endoscopic approach
34	Evaluate, investigate, and operate on patients with benign or malignant mandibular and maxillary lesions
35	Evaluate, investigate, and operate on patients with benign or malignant orbital lesions
36	Indicate and perform fine needle aspiration with or without ultrasound guidance
37	Indicate and perform ultrasound evaluation of cervical structures as a complementary diagnostic method
38	Evaluate, investigate, and operate on patients with benign or malignant paranasal sinus lesions
39	Evaluate, investigate, and operate on patients with skull base surgical conditions
40	Evaluate, investigate, and operate on patients with malignant laryngeal lesions through partial laryngectomy
41	Evaluate, investigate, and operate on patients with malignant laryngeal lesions through total laryngectomy with appropriate vocal rehabilitation
42	Evaluate, investigate, and operate on patients requiring selective neck dissection
43	Evaluate, investigate, and operate on patients requiring classic or modified radical neck dissection
44	Participate in the coordination, organization, and execution of surgical days
45	Participate in and lead educational or administrative activities and clinical meetings
46	Develop a personal learning and professional development plan

## Discussion

The evolution of Head and Neck Surgery as a specialized field in Brazil has a documented trajectory spanning over four decades. The discipline achieved formal recognition as a distinct specialty on July 2nd, 1980, followed by official acknowledgment from the Federal Council of Medicine less than two years later on March 3rd, 1982. This milestone was quickly succeeded by establishing dedicated Medical Residency Programs on September 27th of that same year, institutionalizing the pathway for specialized training in this field.^7^

The contemporary certification process for Head and Neck Surgery specialists follows multiple pathways. The primary route requires completion of an accredited Medical Residency program in either General Surgery or Otolaryngology, followed by a two-year specialized training period in Head and Neck Surgery through programs recognized by the National Medical Residency Commission or equivalent internships acknowledged by the respective Specialty Society.^7^

For practitioners seeking certification without formal specialized training but require substantially more extended periods of documented professional experience. These include demonstrating four years of dedicated practice in Head and Neck Surgery following training in the prerequisite specialties (General Surgery of Otolaryngology) of six years.^7^

The educational framework centered on competencies establishes standardized performance benchmarks as its cornerstone, fundamentally striving to ensure every trainee demonstrates predetermined proficiency thresholds before program completion.^8^ In this educational paradigm, specific abilities and demonstrable skills serve as the foundation for the entire medical curriculum architecture, functioning as discrete yet interconnected building blocks that collectively define professional readiness.^9^

The International Federation of Head and Neck Oncologic Societies has established comprehensive proficiency milestones for specialized fellowship training, encompassing both 1–2 years of clinical immersion and successful fulfillment of all Global Online Fellowship (GOLF) program components.^10^ This framework identifies several essential domains: diagnostic expertise (including advanced endoscopic evaluation and radiological interpretation for accurate tumor assessment and staging); clinical decision-making capacity to determine optimal intervention strategies; surgical autonomy in performing fundamental procedures (encompassing various neck dissection techniques, salivary gland interventions, oral cavity cancer management, thyroid/parathyroid operations, complex laryngeal/pharyngeal/sinus approaches, and interventions for cutaneous, soft tissue, and osseous neoplasms); effective participation within interdisciplinary tumor boards addressing complex cases; and comprehensive understanding of non-operative and adjunctive treatment modalities.^10^ Upon completion of specialized Head and Neck Surgery training, residents and fellows must demonstrate practical application of these established competencies, reflecting mastery of specialized knowledge, technical proficiency, professional conduct, and advanced problem-solving capabilities when confronting highly complex clinical scenarios.

## Conclusion

This research-driven consensus identifies the essential proficiencies that should be attained following completion of specialized Head and Neck Surgery training programs. The established competency framework encompasses both routine and complex surgical interventions that specialists will regularly perform throughout their professional careers.

## ORCID IDs

Marco Aurélio Vamondes Kulcsar: 0000-0002-4751-0476, Luiz Paulo Kowalski: 0000-0002-0481-156X, Fernando Luiz Dias: 0000-0003-1000-7436, Bruna Carteiro Silva: 0009-0006-3444-885X

## Funding

None.

## Declaration of competing interest

The authors declare no conflicts of interest.
